# Economic growth and global warming effects on electricity consumption in Spain: a sectoral study

**DOI:** 10.1007/s11356-022-22312-5

**Published:** 2022-08-06

**Authors:** Maria del P. Pablo-Romero, Antonio Sánchez-Braza, Daniel González-Jara

**Affiliations:** Department of Economic Analysis and Political Economy, Faculty of Economics and Business Sciences, Universidad de Sevilla, Av. Ramon y Cajal 1, 41018 Seville, Spain

**Keywords:** Electricity consumption, Economic growth, Economic sectors, Global warming, Spain, Heating and cooling degree days (HDD & CDD)

## Abstract

This paper analyzes the effect of certain factors on electricity consumption in Spain at a sectoral level. An electricity consumption function has been estimated by using panel data, depending on gross value added (GVA), temperatures, capitalization, and human capital. This function is obtained for total productive electricity consumption and for the agricultural, construction, industrial, service, and public administration sectors, referring to the 17 Autonomous Communities of Spain for the 2000–2013 period. The obtained results show important sectoral differences in the effect that GVA has on electricity consumption, indicating a positive and increasing effect of temperatures above 22 °C in the total economy and in the tertiary sector, and a negative effect of temperatures below 18 °C in some sectors. These results may indicate that global warming may induce an electricity demand growth in Spain, especially related to cooling needs. The results also highlight the positive effects of capitalization in all sectors, and the negative effects of human capital, except for the public administration sector. In this context, it may be appropriate to carry out policies that mitigate this consumption growth, reinforcing energy efficiency measures, and human capital investments.

## Introduction

Signatory countries of the 2015 Paris Agreement were committed to adopting national targets to reduce greenhouse gas (GHG) emissions (UNFCCC 2015). In order to achieve these targets, the control of energy consumption from non-polluting sources has acquired great importance. In this sense, according to the US Energy Information Administration (2016), energy consumption analysis is set at the center of the political and economic debate on climate change, since most GHG emissions are caused by fossil fuel consumption. In this sense, according to the International Energy Agency (IEA 2015), energy is responsible for two-thirds of GHG and 80% of CO_2_ emissions.

All energy sectors are relevant to the reduction of CO_2_ emissions. However, the electricity sector has special relevance, because it was responsible for 38% of worldwide energy-related CO_2_ emissions in 2018 (IEA 2019). Therefore, the analysis of electricity production and consumption evolution is relevant in order to define policies aimed at achieving the national targets. In this vein, it is worth noting that between 2010 and 2016, emissions from electricity production grew by 7% worldwide (IEA 2018). Nevertheless, in both the Americas and Europe, increases in renewable share of the energy mix and improvements in fossil generation efficiency led to emission reductions. In fact, the GHG emission intensity of total electricity generation in the EU-27 decreased from 510 g CO_2_e/kWh to 281 g CO_2_e/kWh from 1990 to 2018 (European Environment Agency 2021).

In Europe, it is worth highlighting Spain for its lower proportion of emissions generated by the electricity sector. The IEA (2018) report shows that, while at the European level these emissions accounted for 34.5% of total CO_2_ emissions in 2016, these were 27.7% in Spain. The GHG emission intensity of total electricity generation was 276 g CO_2_e/kWh in Spain in 2018, lower than that by the EU-27 (European Environment Agency 2021). Therefore, it is particularly interesting to analyze the situation of the electricity sector in this country.

Some previous studies have analyzed the Spanish electricity sector from different perspectives. For example, that by Del Río (2008) on the effects of feed-in-tariff premiums on electricity production and that by Burgos-Payán et al. (2013) on the costs and benefits of electricity from renewable energies. Along with them, other studies have focused on the effect of economic growth on Spanish electricity consumption. In this vein, the studies by Ciarreta and Zarraga (2010) and Blázquez et al. (2013) focused on the residential sector. These latter studies are of special interest, since numerous previous studies (see “Literature review”) show a clear link between both variables, which can condition the final growth of electricity consumption.

In the context of global warming, the analysis of this study is interesting. According to previous research, electricity consumption may be affected by rising temperatures in Spain (Ministry of the Environment 2006). This country belongs to the Mediterranean area, a territory particularly vulnerable to the global warming process, where temperature increases above the European average are expected (Ministry of the Environment 2006; European Environment Agency 2017).

The aim of this paper is to analyze the effect of economic growth on Spanish electricity consumption, at a sectoral level, in a global warming context. To this end, panel data are used to estimate an electricity consumption function that depends on the GVA, its squared and cube values, production factors, and temperatures. The data panel refers to the 17 Spanish Autonomous Communities for the period from 2000 to 2013, years for which there is enough statistical information available. The electricity consumption function is estimated for the total productive electricity consumption (without residential electricity consumption) and for the following sectors: agriculture, construction, industry, service, and public administration. Therefore, this study enlarges the previous literature by analyzing the non-linear relationship between electricity consumption and economic growth from a sectoral perspective. Nevertheless, to our knowledge, this study goes beyond previous studies. Firstly, it refers to Spain, for which there are no previous studies. Secondly, it considers the human and physical capital productive factors as additional factors that influence energy consumption, in non-linear economic growth effects analysis, which has not been considered previously. Finally, this study analyzes the effect of temperatures on electricity consumption by sectors. The results of this study can help to design energy policy measures aimed at achieving the Spanish national targets related to energy and emissions.

This study is structured in the following sections: after this introduction, the previous literature is reviewed in “Literature review”; “Data” presents the databases used; “Methodology” describes the methodology used; “Results” presents the results, while “Discussion” discusses these results; and, finally, “Conclusion and policy implications” concludes the study.

## Literature review

Previous studies that analyze the effect of certain factors on energy consumption in general, and on electricity consumption in particular, are very broad. Below, previous literature considered relevant for this study is presented.

### Energy consumption and economic growth: the energy-EKC hypothesis

The relationship between energy consumption and economic growth was analyzed for the first time by Kraft and Kraft (1978). Since then, numerous studies have focused on this topic. An extensive review of these studies has been carried out previously in Tiba and Omri (2017). These reviews indicate that a large part of the studies show that economic growth causes, and positively affects, energy consumption.

Following this line of analysis, some studies have shown that this relationship is not linear, but it depends on income level evolution. The study by Suri and Chapman (1998) may be considered one of the pioneers in analyzing this non-linear relationship, in what has come to be called the energy Kuznets curve (energy-EKC).

The EKC hypothesis states that, initially, low-income countries have low pollution levels, which grow as their income level does. However, once a certain level of income has been reached, economic growth will cause a pollution decrease. In short, the relationship between the variables has an inverted U shape. Following this hypothesis, some researchers have taken the name of energy-EKC (Dong and Hao 2018) to refer to the inverted U shape observed in the relationships between energy consumption and income level growth.

Among the studies that have found evidence in favor of the energy-EKC hypothesis, those by Nguyen-Van (2010), Yoo and Lee (2010), and Sbia et al. (2017) may be cited. Among those that do not find said evidence, those by Luzzati and Orsini (2009), Zilio and Recalde (2011), and Pablo-Romero and De Jesús (2016) may be cited. Nevertheless, these last studies observe non-linear relationships between the considered variables. Thus, the previous studies’ results highlight the convenience of considering non-linear relationships between the variables.

### Energy consumption and economic growth: sectoral studies

The sectoral analysis of the relationships between energy consumption and economic growth is highly relevant. There are numerous previous studies that show differences in the relationships between these variables when considering different economic sectors. Among these studies, those by Costantini and Martini (2010), Zhang and Xu (2012), Rahman et al. (2015), and recently Bal et al. (2022) might be pointed out.

Nevertheless, studies that simultaneously consider the non-linear relationships between these variables and a sectoral perspective are scarce. Some studies have referred to specific sectors. Among them, for instance, the studies by Liu et al. (2016), Pablo-Romero and Sánchez-Braza (2017), and Pablo-Romero et al. (2019) refer to the residential sector; those by Katircioglu (2014) and Pablo-Romero et al. (2017a) refer to tourism; and those by Lin and Du (2015), Pablo-Romero et al. (2017b), and Rehermann and Pablo-Romero (2018) to the transport sector.

However, very few studies have compared the effect of several sectors simultaneously in a context of non-linearity between the variables. Among these, the early study by Judson et al. (1999), which referred to 69 countries, may be cited. Likewise, the studies by Lescaroux (2011), which referred to 101 countries; the study by Burke and Csereklyei (2016), which referred to 132 countries; and that by Liddle (2017), which referred to the 50 USA states, may also be highlighted. Finally, it is also worth noting the study by Howarth et al. (2017) which referred to Gulf Cooperation Council States. In general, all these studies show clear elasticity of energy consumption with heterogeneity regarding income among the sectors studied. This justifies the sectoral disaggregation in the present study.

### Energy consumption and economic growth: influence of other factors

The inclusion of additional factors in the study of the relationship between economic growth and energy consumption is frequent in the literature. In this sense, Costantini and Martini (2010) consider that the inclusion of these factors is convenient. Thus, different variables have been considered in previous studies, depending on the scope of the study. Among these variables, for example, trade openness has been included in Kasman and Duman (2015), population density in Holden and Norland (2005), and urbanization in Dujardin et al. (2014). Taking into account their relative novelty, the study of the sectoral perspective, and the climate change context, two additional variable groups will be considered in the current study.

The first group of variables to take into account refers to the combination of productive factors used in the production process. Numerous previous studies have observed that both physical and human capital have complementarity or substitutability relationships with respect to energy consumption. Among them, the studies by Lin and Wesseh (2013), Pablo-Romero and Sánchez-Braza (2015), and Li and Lin (2016) may be cited. Additionally, in the study by Román-Collado and Colinet (2018), the effect of labor productivity on energy consumption is analyzed.

Taking into account this fact, some recent studies have included the physical and/or human capital stock as additional energy consumption explanatory variables. Among them, the studies by Fang and Chen (2017), Salim et al. (2017), Chen and Fang (2018), and Fang and Yu (2020) may be cited, although none of them consider the non-linear relationships between economic growth and energy consumption. Only two studies that, as far as we know, also consider these non-linear effects are those by Balaguer and Cantavella (2018) and by Fang and Wolski (2021) (although only the human capital effects are analyzed in these studies). In addition, recently, the study by Hanif et al. (2020) evaluates the effect of human capital and technology innovation on renewable and non-renewable energy consumption.

The second group of variables is introduced in the model to take into account the effect of temperatures on electricity consumption. According to Burke et al. (2015), it is expected that global warming will directly affect productive income. Therefore, it is relevant to deepen the knowledge of the effects it has on the productive sector electricity demand.

Numerous econometric studies incorporate temperature in the energy consumption analysis. Auffhammer and Mansur (2014) carry out a review of these works, indicating that they mainly focus on the households’ electricity consumption with a microeconomic perspective. Nevertheless, there are also an increasing number of macroeconomic studies referring to the residential sector, for example, that by Auffhammer (2018) and Nie et al (2018).

Despite these macroeconomic studies, there are few macroeconomic studies focused on other economy sectors. In this vein, there are very few studies jointly analyzing temperature and productive effects on electricity consumption (Ge et al. 2017) and considering the temperature effects by economic sectors (Fan et al. 2015). In this last study, the authors conclude that although the results indicate that the temperature changes have more impact on the household and tertiary sectors, the primary and the secondary sectors are also influenced by them. Likewise, some studies have highlighted the effect of temperatures on several productive sectors, especially on the commercial ones (Hirano and Fujita 2012). Therefore, it is interesting to study also the effect of temperature on the electricity demand of these productive sectors.

Focusing on Spain, there are few studies analyzing the temperature effects on electricity consumption. To our knowledge, these studies focus on the residential sector (Blázquez et al. 2013; Pablo-Romero et al. 2021), on households (Romero-Jordán et al. 2014), on total electricity consumption (Moral-Carcedo and Vicens-Otero 2005), and on the tourism sector (Pablo-Romero et al. 2017a). Additionally, the study by Moral-Carcedo and Pérez-García (2015) considers the effect on different sectors, although they do not consider the possible non-linear effect of CDD and HDD.

Taking into account the previous studies, this study enlarges the existing literature by analyzing the non-linear relationship between electricity consumption and economic growth from a sectoral perspective. In this vein, there is little empirical evidence to date, and none related to Spain. Likewise, this study considers the human and physical capital productive factors and integrates them into a non-linear economic growth effects analysis. To our knowledge, this has not been considered previously. Finally, this study analyzes the non-linear effect of temperatures on electricity consumption by sectors, which is also a novelty in the literature.

## Data

This study analyzes the electricity consumption of the 17 Spanish Autonomous Communities in the 2000–2013 period. The study refers to total productive electricity consumption and to the following sectoral consumption: agriculture, industry, construction, service, and public administration. Total productive electricity consumption refers to the total electricity consumption, excluding residential consumption. The sectoral aggregation detail is shown in Table 1.

**Table 1:** Sectoral aggregation detail

Sector	CNAE 2009	Excluded subsectors	Additional included subsectors
Agriculture	01 to 03		
Industry	05 to 39	23.1, 23.5, 24.4, 24.46, 24.53, 24.54, 33.15	
Construction	41 to 43		
Service	45 to 82		31.01, 31.02, 31.03, 31.09, 33.12
Public administration	85 to 99	85.51, 85.52, 85.53, 85.56, 85.59	36 to 39

### Data on electricity consumption

Data related to electricity consumption are from the annual electrical statistics of the Spanish Ministry of Energy (Ministerio de Industria, Energía y Turismo 2019). Data are expressed in megawatt hours (Mwh) per thousand employed, converted into natural logs.

**Fig. 1:**
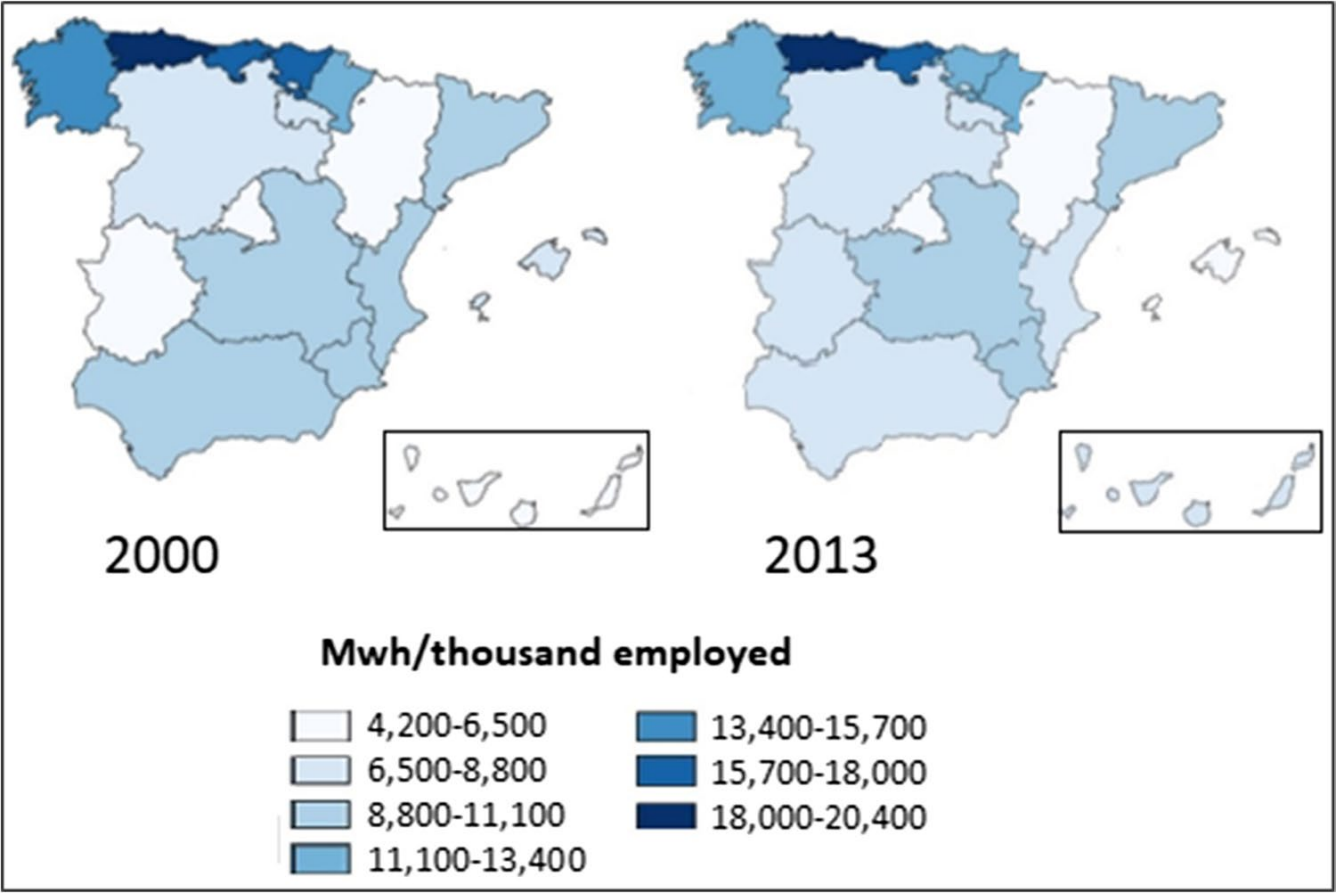
Total productive electricity consumption in Spain by Autonomous Communities: 2000 and 2013 (MWh per thousand employed). Source: own elaboration from Ministerio de Industria, Energía y Turismo (2019)

Figure 1 shows the total productive electricity consumption per thousand employed in the Spanish Autonomous Communities in 2000 and 2013. Noticeable regional differences with relatively small changes between periods may be observed. Therefore, although greater amounts of electricity are consumed in 2013, the regional composition has not changed significantly.

**Fig. 2:**
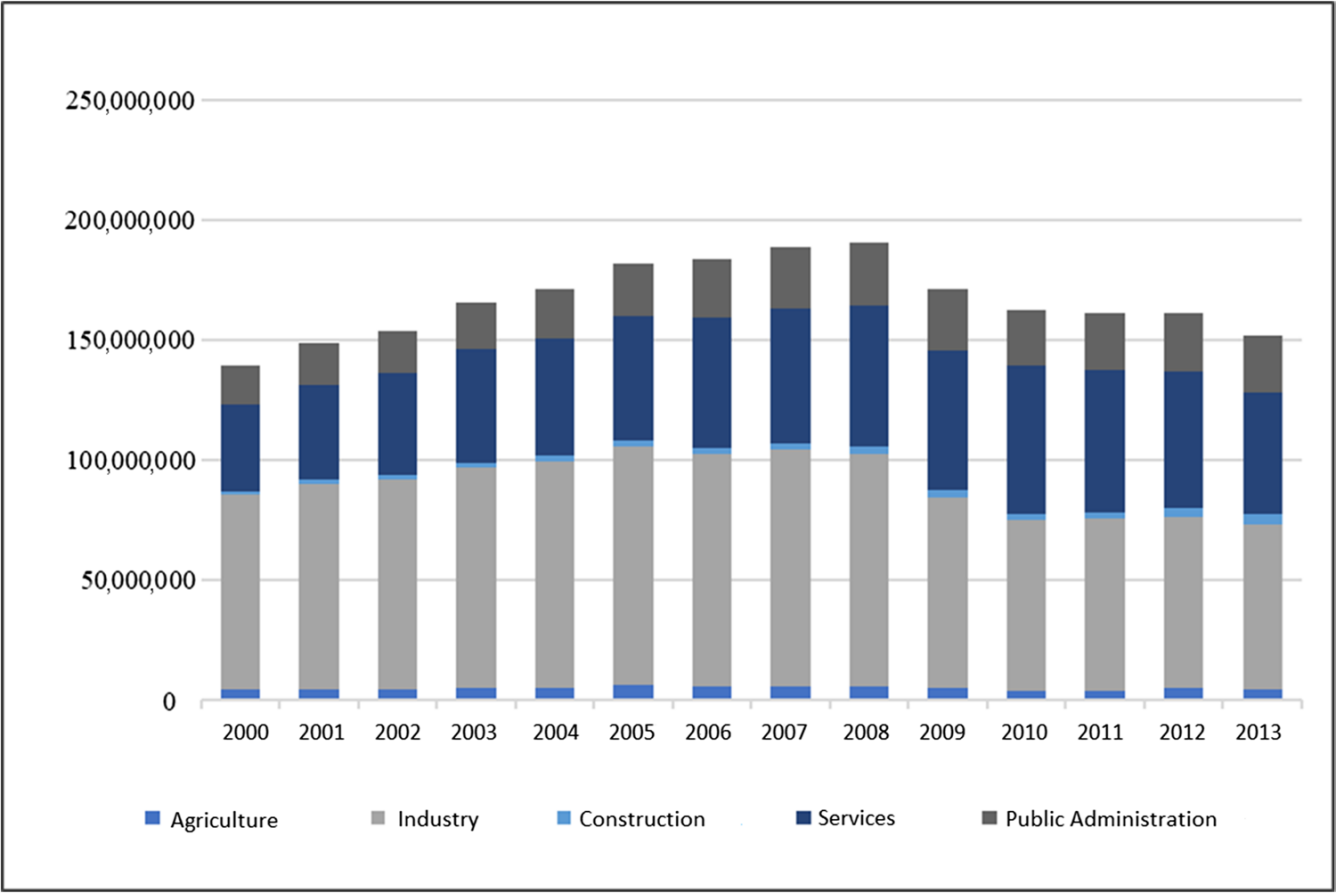
Total productive electricity consumption by sectors: 2000–2013 (MWh). Source: own elaboration from Ministerio de Industria, Energía y Turismo (2019)

Figure 2 shows the electricity consumption trend by productive sectors. Total productive electricity consumption grew until 2008, decreasing later. In 2000, sectors with the highest electricity consumption participation on total productive value were industry (58%), service, and public administration (38%, jointly). In 2013, these sectors were also the ones with the highest participation. Nevertheless, industry sector participation decreased until reaching 49%, while service and public administration increased.

### Data on GVA and productive factors

Data on total and sectoral production are obtained from the Spanish regional accounting offered by the National Statistics Institute (INE 2019). Gross value added (GVA) at 2010 basic prices, in millions of euros per thousand employees logs, is used.

Data on physical and human capital stock are from the Instituto Valenciano de Estudios Económicos database (IVIE 2019). Productive physical capital value is considered. Data are expressed in millions of euros per thousand employees, in logs. Human capital is expressed in terms of persons with higher studies per thousand employees, in logs.

### Data on temperatures

Data from the Agencia Estatal de Meteorología (2019) have been used to calculate the temperature variables. Specifically, data on average daily temperatures from the main provinces’ meteorology stations have been used. From these data, CDD (cooling degree days) and HDD (heating degree days) variables have been calculated for the Spanish provinces and autonomous communities.

CDD and HDD have been extensively used in recent literature, for example, in Fan and Hyndman (2011), Blázquez et al. (2013), and Mohammadi and Ram (2017).

The CDD variable is calculated as follows. Firstly, it is necessary to calculate the CDD variable for each day in a year, as in Eq. (1):

1 $$\mathrm{CDDi}=(1-\upgamma ) (\mathrm{Ti}-\mathrm{Tb})$$

where Ti is the average temperature registered in a Spanish province on day *i*, and Tb is a base or reference temperature. Tb is the threshold temperature. Above this value, it is necessary to turn on the cooling devices. If the temperature is higher than Tb, then *γ* = 0, and the variable CDD takes the value equal to (Ti-Tb) on day *i*. If the average temperature is lower than Tb, then *γ* = 1 and CDD = 0 for that day.

Once the daily value is calculated, the annual CDD value for each Spanish province is calculated as the sum of the daily values for each province. Finally, the CDD for the Spanish autonomous communities is calculated as the weighted average (according to population) of the provinces’ CDD values.

Likewise, the HDD variable is calculated as follows. The HDD variable for each day in a year is calculated for each Spanish province, as in Eq. (2):

2 $$\mathrm{HDDi}=(1-\upgamma ) (\mathrm{Tb}-\mathrm{Ti})$$

In this case, if the daily average temperature falls below the reference value (Tb), it is necessary to turn on the heating devices. Then, analytically, *γ* = 0, and HDD = (Tb—Ti). On the contrary, if it does not fall below that value, *γ* = 1 and HDD = 0.

As before, the annual value is calculated from the sum of the daily values. Likewise, the HDD for each Spanish autonomous community is calculated as the weighted average (according to population) of the provinces’ HDD values.

The reference temperatures for calculating the CDD and HDD variables are 22 °C and 18 °C, respectively. According to Blázquez et al. (2013) and Pablo-Romero et al. (2021), these values can be considered appropriate for Spain. In this study, the CDD and HDD values have been converted into logs.

### Main descriptive statistics

The main descriptive statistics of variables are shown in Table 2. The overall statistics refer to the panel sample, the within statistics to the variation from each community’s average, and the between ones to the variation from time’s average. As observed, in general, the typical standard deviation is higher across the Spanish Autonomous Communities than it is across time.

**Table 2:** Descriptive statistics

Electricity consumption per employee in log		Mean	Std. dev	Min	Max	Observations
Total productive sectors	Overall	9.088	0.400	7.993	9.975	*N* = 238 *n* = 17 *T* = 14
	Between		0.400	8.311	9.875	
	Within		0.093	8.316	9.338	
Agriculture sector	Overall	8.435	0.810	5.997	10.832	*N* = 238 *n* = 17 *T* = 14
	Between		0.753	6.860	9.432	
	Within		0.345	7.549	10.410	
Construction sector	Overall	7.008	0.710	5.069	9.149	*N* = 238 *N* = 17 *T* = 14
	Between		0.485	5.649	7.470	
	Within		0.530	5.414	8.928	
Industrial sector	Overall	10.174	0.619	8.657	11.654	*N* = 238 *N* = 17 *T* = 14
	Between		0.611	9.066	11.462	
	Within		0.174	9.035	11.447	
Public administration sector	Overall	8.284	0.421	6.503	8.965	*N* = 238 *N* = 17 *T* = 14
	Between		0.377	7.097	8.822	
	Within		0.207	6.908	8.854	
Service sector	Overall	8.732	0.378	7.309	9.438	*N* = 238 *N* = 17 *T* = 14
	Between		0.343	7.477	9.061	
	Within		0.179	7.371	9.309	
GVA per employees in log		Mean	Std. dev	Min	Max	Observations
Total productive sectors	Overall	10.776	0.089	10.545	10.994	*N* = 238 *N* = 17 *T* = 14
	Between		0.078	10.636	10.918	
	Within		0.046	10.685	10.899	
Agriculture sector	Overall	10.242	0.220	9.738	10.780	*N* = 238 *N* = 17 *T* = 14
	Between		0.171	9.961	10.524	
	Within		0.144	9.893	10.609	
Construction sector	Overall	10.803	0.166	10.365	11.298	*N* = 238 *N* = 17 *T* = 14
	Between		0.114	10.590	11.074	
	Within		0.124	10.544	11.138	
Industrial sector	Overall	11.000	0.143	10.661	11.367	*N* = 238 *N* = 17 *T* = 14
	Between		0.103	10.822	11.162	
	Within		0.101	10.778	11.225	
Public administration sector	Overall	10.567	0.052	10.448	10.689	*N* = 238 *N* = 17 *T* = 14
	Between		0.033	10.501	10.621	
	Within		0.040	10.464	10.660	
Service sector	Overall	10.862	0.102	10.587	11.120	*N* = 238 *N* = 17 *T* = 14
	Between		1.393	1.887	6.355	
	Within		0.495	1.107	5.893	
Productive physical capital per employees in log		Mean	Std. dev	Min	Max	Observations
Total productive sectors	Overall	11.374	0.177	10.993	11.806	*N* = 238 *N* = 17 *T* = 14
	Between		0.129	11.184	11.602	
	Within		0.125	11.162	11.670	
Agriculture sector	Overall	11.212	0.454	10.128	12.572	*N* = 238 *N* = 17 *T* = 14
	Between		0.420	10.668	12.056	
	Within		0.197	10.672	11.728	
Construction sector	Overall	11.553	1.486	8.800	15.511	*N* = 238 *N* = 17 *T* = 14
	Between		1.472	9.224	14.807	
	Within		0.401	11.045	12.460	
Industrial sector	Overall	12.083	1.215	9.822	14.462	*N* = 238 *N* = 17 *T* = 14
	Between		1.235	10.147	14.100	
	Within		0.184	11.758	12.556	
Public administration sector	Overall	11.341	0.203	10.765	11.733	*N* = 238 *N* = 17 *T* = 14
	Between		0.189	10.967	11.609	
	Within		0.086	11.114	11.535	
Service sector	Overall	11.008	0.179	10.672	11.465	*N* = 238 *N* = 17 *T* = 14
	Between		0.164	10.755	11.348	
	Within		0.081	10.851	11.276	
Human capital per employees in log		Mean	Std. dev	Min	Max	Observations
Total productive sectors	Overall	5.959	0.235	5.119	6.549	*N* = 238 *N* = 17 *T* = 14
	Between		0.197	5.463	6.332	
	Within		0.136	5.614	6.307	
Agriculture sector	Overall	5.155	0.591	3.852	7.136	*N* = 238 *N* = 17 *T* = 14
	Between		0.455	4.362	6.475	
	Within		0.392	3.891	6.303	
Construction sector	Overall	5.469	0.353	4.449	6.246	*N* = 238 *N* = 17 *T* = 14
	Between		0.231	4.982	5.824	
	Within		0.273	4.855	6.189	
Industrial sector	Overall	5.852	0.333	4.629	6.478	*N* = 238 *N* = 17 *T* = 14
	Between		0.292	5.184	6.253	
	Within		0.173	5.297	6.316	
Public administration sector	Overall	5.931	0.199	5.363	6.476	*N* = 238 *N* = 17 *T* = 14
	Between		0.188	5.648	6.415	
	Within		0.078	5.647	6.134	
Service sector	Overall	6.186	0.268	5.368	6.847	*N* = 238 *N* = 17 *T* = 14
	Between		0.241	5.638	6.665	
	Within		0.130	5.850	6.575	
Temperature		Mean	Std. dev	Min	Max	Observations
HDD	Overall	6.289	1.263	0.969	7.771	*N* = 238 *N* = 17 *T* = 14
	Between		1.274	2.539	7.647	
	Within		0.249	4.719	7.622	
CDD	Overall	4.159	1.442	− 0.916	6.579	*N* = 238 *N* = 17 *T* = 14
	Between		1.393	1.887	6.355	
	Within		0.495	1.107	5.893	

## Methodology

### The model

The initial theoretical modeling in this study is the following electricity consumption function:

3 $${\mathrm{E}}_{\mathrm{it}}=\mathrm{f }({\mathrm{Y}}_{\mathrm{it}},{\mathrm{K}}_{\mathrm{it}} , {\mathrm{H}}_{\mathrm{it}}, {\mathrm{temp}}_{\mathrm{it}})$$

where *E* is the electricity consumption per employee, *Y* represents the GVA per employee, *K* is the capital stock per employee used in production, *H* is the human capital indicator described above, temp refers to some temperature indicator, *i* indicates the Spanish Autonomous Communities, and *t* is a time variable.

Therefore, electricity consumption depends on the productive level, productive factor combination, and temperatures. Two considerations related to this model specification should be noted. Firstly, in some previous recent studies (such as, for example, Balaguer and Cantavella 2018; Chen and Fang 2018; Fang and Chen 2017; and Salim et al. 2017), it has been indicated that electricity consumption depends not only on the level of production level, but also on the way in which the productive factors are combined in the production process. Therefore, the aforementioned studies include physical and/or human capital as additional explanatory variables. In this study, that research line is followed and both variables are included in the electricity consumption function specification. Secondly, previous studies consider also that temperatures may influence energy or electricity consumption (Gam and Rejeb 2012; Lee and Chiu 2011, among others). In this study, this research line is followed, and therefore temperature variables have been included in the model.

In addition to these considerations, two other questions may be taken into account. On the one hand, many previous studies have observed that the relationship between production and energy consumption is not linear (Nguyen-Van 2010; Pablo-Romero and Sánchez-Braza 2017; Sbia et al. 2017; Suri and Chapman 1998, among others). Thus, when specifying the energy consumption function, not only the production variable has been considered, but also the squared and cubed value of production. This consideration has given rise to what has come to be called the energy-EKC hypothesis (Dong and Hao 2018).

On the other hand, the effect of temperatures on energy consumption has also been considered non-linear, in a double sense. Firstly, previous studies have shown that the temperatures’ effect on energy consumption varies depending on whether the temperatures are low or high. That is to say, it varies according to the heating or refrigeration needs, generating greater energy consumption when both situations appear. As a consequence, two variables have been used, instead of one, to analyze the temperatures’ effect on electricity consumption. Usually, in previous studies (Blázquez et al. 2013; Fan and Hyndman 2011; Mohammadi and Ram 2017, for example), these variables are the CDD and HDD.

Secondly, other authors have additionally considered that these two variables may also have a non-linear effect on electricity consumption. Thereby, in these studies (for example, in Boyd 2014), the CDD and HDD squared values have also been included to analyze this non-linear temperature effect. In this study, this last specification has been considered.

Taking into account the above considerations, the electricity consumption function in this study is specified as follows:

4 $$ln{E}_{it}\hspace{0.33em}\hspace{0.33em}=\hspace{0.33em}\hspace{0.33em}\hspace{0.33em}\hspace{0.33em}{A}_{it}+{\beta }_{1}\mathit{ln}{Y}_{it}+{\beta }_{2}(\mathit{ln}{Y}_{it}{)}^{2}+{\beta }_{3}(\mathit{ln}{Y}_{it}{)}^{3}+{\beta }_{4}\mathit{ln}{K}_{it}+{\beta }_{5}\mathit{ln}{H}_{it}+{\beta }_{6}\mathit{ln}HD{D}_{it}+{\beta }_{7}(\mathit{ln}HD{D}_{it}{)}^{2}+{\beta }_{8}\mathit{ln}CD{D}_{it}+{\beta }_{9}(\mathit{ln}CD{D}_{it}{)}^{2}+{e}_{it}$$

where *A* represents the sum of the temporary and individual effect, and *e* is the error term.

The estimated coefficients related to production (variable *Y*) inform about the relationship between electricity consumption and production. In this sense, as Pablo-Romero et al. (2021) state, it is possible to find different types of relationships. The following non-linear relationships should be noted:

- *β*_1_ > 0, *β*_2_ > 0, and *β*_3_ = 0, progressively increasing relationship.
- *β*_1_ > 0, *β*_2_ < 0, and *β*_3_ = 0, U-shaped inverted relationship.
- *β*_1_ < 0, *β*_2_ > 0, and *β*_3_ = 0, U-shaped relationship.
- *β*_2_^2^-3*β_3_*β*_1_ > 0 and *β*_3_ > 0, N-shaped relationship.
- *β*_2_^2^-3*β_3_*β*_1_ > 0 and *β*_3_ < 0, N-shaped inverted relationship.

Likewise, the *β*_6_ and *β*_7_ coefficients and the *β*_8_ and *β*_9_ ones inform about the relationships between electricity consumption and that of cold and heating temperature, respectively. For the two coefficient pairs, if both are positive, then there is an increasing relationship between both variables. If the second coefficient is equal to zero, the relationship is linear. If the first coefficient is positive and the second is negative, there is a positive but decreasing relationship. Finally, if the first coefficient is negative and the second is positive, there is a negative but growing relationship. Thus, the study of these coefficients will inform about the CDD and HDD non-linear effect on electricity consumption.

### Estimation procedure

Two considerations have been taken into account when estimating Eq. (4). Firstly, the introduction of some variables in quadratic or cubic terms may generate multicollinearity problems. However, according to Jaccard and Turrisi (2003), these problems can be mitigated by centering their values. Therefore, variables in Eq. (4) have been transformed in terms of geometric mean deviations. This transformation implies that *β*_1_, *β*_6_, and *β*_8_ coefficients now represent the electricity consumption elasticity with respect to production, to HDD, and to CDD, at the sample center point.

Secondly, once the variables have been transformed, the stochastic nature of the series has been studied. Table 3 shows Pesaran’s cross-sectional (CD) dependence test results (Pesaran 2004). For all series, the Pesaran CD test results indicate that the cross-sectional independence null hypothesis is rejected. Thus, a second-generation panel unit root test should be performed. In this study, the cross-sectionally augmented Im, Pesaran, and Shin panel unit root tests (CIPS) by Pesaran (2007) are used.

**Table 3:** Pesaran’s CD tests

Variables	CD test
*E*	6.21***
*Y*	34.27***
*Y*^2^	2.45**
*Y*^3^	27.99 ***
*K*	42.83***
*H*	41.13***
HDD	28.85***
HDD^2^	1.84*
CDD	27.79***
CDD^2^	1.67*

*** denotes significance at the 1% level, ** at the 5% level, and * at the 10% level

Table 4 shows the CIPS test results for series in levels and first differences when considering intercept and trend. The results indicate that all variables cannot be considered stationary at a 5% significance level, but are I(1). Therefore, estimating in first differences may be appropriate. According to Stern et al. (2017), expressing Eq. (4) in first differences is like expressing the energy-EKC in long-run growth rates terms. Therefore, the variables have also been converted into first differences.

**Table 4:** CIPS test

Variables	Level	First differences
	Intercept and trend	Intercept and trend
*E*	− 2.702*	* − *3.362***
*Y*	* − *2.435	* − *4.286***
*Y*^2^	* − *0.612	* − *3.137 ***
*Y*^3^	* − *2.035	* − *2. 771**
*K*	* − *2.290	* − *3.179***
*H*	* − *1.889	* − *3.379***
HDD	* − *3.008**	* − *4.249***
HDD^2^	* − *2.968**	* − *4.060***
CDD	* − *2.776*	* − *3.323***
CDD^2^	* − *3.002**	* − *3.782 ***

*t*-bar statistics *** denotes significance at the 1% level, ** at the 5% level, and * at the 10% level. Logs calculated with an iterative process from 0 to 3 based on *F*-joint test. The truncated version of the test is applied

Taking into account both variable transformations, Eq. (4) is now expressed as follows:

5 $$\Delta \overline{\mathit{ln}{E}_{it}}\hspace{0.33em}\hspace{0.33em}=\hspace{0.33em}\hspace{0.33em}\hspace{0.33em}\hspace{0.33em}{\delta }_{t}+{\beta }_{1}\hspace{0.33em}\Delta \overline{\mathit{ln}{Y}_{it}}+{\beta }_{2}\hspace{0.33em}\Delta (\overline{\mathit{ln}{Y}_{it}}{)}^{2}+{\beta }_{3}\hspace{0.33em}\Delta (\overline{\mathit{ln}{Y}_{it}}{)}^{3}+{\beta }_{4}\hspace{0.33em}\Delta \overline{\mathit{ln}{K}_{it}}+{\beta }_{5}\hspace{0.33em}\Delta \overline{\mathit{ln}{H}_{it}}+{\beta }_{6}\hspace{0.33em}\Delta \overline{\mathit{ln}HD{D}_{it}}+{\beta }_{7}\hspace{0.33em}\Delta (\overline{\mathit{ln}HD{D}_{it}}{)}^{2}+{\beta }_{8}\hspace{0.33em}\Delta \overline{\mathit{ln}CD{D}_{it}}+{\beta }_{9}\hspace{0.33em}\Delta (\overline{\mathit{ln}CD{D}_{it}}{)}^{2}+{e}_{it}$$

where $\Delta \overline{{A }_{it}}=$ δt, Δ indicates that variables are in first differences terms, and the line on the variables indicates they are in terms of geometric mean deviations.

Finally, autocorrelation and homoscedasticity have also been analyzed to determine the estimated model of (5). Therefore, Wooldridge (2002) and the Wald test for homoscedasticity (Greene 2000) have been performed. Taking into account the test results, Eq. (5) is estimated by using the feasible generalized least squares (FGLS) method when controlling for autocorrelation, heteroscedasticity, and contemporaneous correlation.

## Results

Tables 5, 6, 7, 8, 9 and 10 show the estimate results of Eq. (5), Table 5 shows the results when referring to all productive sectors, and Tables 6, 7, 8, 9 and 10 show the results for the considered sectors. All estimates have been performed by using FGLS and include time dummies.

### Estimate results of total productive sector

Column 3 in Table 5 shows the estimate results of Eq. (5) for total productive sectors. The results indicate non-linear relationships between production and electricity consumption. Specifically, an N-shaped relationship is observed. Thus, although a negative relationship is observed for low production levels, this relationship becomes positive when the production is higher. Therefore, the production increase may provoke an electricity consumption growth.

**Table 5:** Estimate results for total productive sectors

Variable	Coefficient	FGLS	FGLS
*Y*	*β*_1_	0.847***	0. 884***
		(0.152)	(0.127)
*Y*^2^	*β*_2_	− 3.416***	− 3.109***
		(0.739)	(0.649)
*Y*^3^	*β*_3_	15.636***	8.387***
		(4.251)	(4.401)
*K*	*β*_4_	0.738***	0.451***
		(0.131)	(0.133)
*H*	*β*_5_	− 0.153**	− 0.132*** (0.031)
		(0.043)	(0.031)
*HDD*_18_	*β*_6_	− 0.130***	0.004*
		(0.016)	(0.002)
*HDD*_18_^2^	*β*_7_	− 0.018***	
		(0.002)	-
*CDD*_22_	*β*_8_	0.021***	0.005**
		(0.005)	(0.002)
*CDD*_22_^2^	*β*_9_	0.003***	-
		(0.001)	

*** denotes significance at the 1% level, ** at the 5% level, and * at the 10% level

Regarding the temperature variables, it is worth noting the positive and significant value for the CDD coefficient, and the negative and significant value for the HDD one. The positive value for the CDD coefficient indicates that, as the temperature increases above 22 °C, electricity consumption increases. In addition, the positive and significant value associated to the squared CDD variable points out a progressively increasing electricity consumption as temperatures increase. Therefore, as the temperature increases, electricity consumption gradually increases. These values are in line with previous research related to residential energy in Spain, as, for example, in the study by Pablo-Romero et al. (2021). The negative value associated to the HDD variable proves that, as the temperature decreases below 18 °C, electricity consumption is lower. As before, the negative value of the squared HDD variable shows a non-linear behavior. Instead, as the temperature decreases, electricity needs are also decreasing. This negative effect is not observed in the previous studies for the Spanish provinces’ residential energy demand, when not considering the squared values for HDD and CDD, as in Blázquez et al. (2013). This negative effect has not been observed either in the study of residential energy demand in Andalusian municipalities by Pablo-Romero et al. (2021), although the municipalities all belong to the Mediterranean climate area. Therefore, the result has been contrasted by re-estimating Eq. (5) without considering the squared value of the climatic variables. The new estimate results are shown in column 4 in Table 5. Now, both temperature variables are significant and positive, in line with previous studies. The change in sign of the HDD variable when its square value is included in the estimate may be due to the opposite effect that low temperatures can have when they are extreme. Therefore, the negative effect of HDD on electricity consumption may be explained by a fuel substitution caused by temperature falls. In this sense, if the heating needs are greater, petroleum-based fuels, such as gas, may be more efficient or cheaper. In this line, it is worth noting that Fazeli et al. (2016) have pointed out the convenience of taking into account the interfuel substitution as a key factor for projecting energy demand. In this line, it is worth noting that the use of electricity for heating in the residential buildings in Spain was just 5.5% of total buildings, decreasing this percentage for the Spanish continental climate zone, where extreme cold temperatures are present (Cichí et al. 2017).

Finally, the results of the productive variable coefficients are analyzed. The coefficient associated with the stock of capital is positive and significant. Therefore, the physical capital use increases in production lead to an increase in electricity consumption. Along these lines, some previous studies have established the possibility that current capitalization, with a greater tendency to digitalization, may contribute to the reduction of energy consumption by increasing energy efficiency and sectoral changes. However, as pointed out in the study by Lange et al. (2020), the balance seems to be producing additional electricity consumption, predominating the direct effects. On the contrary, the coefficient associated with the human capital variable is negative and significant. Thus, in line with previous studies (Chen and Fang 2018; Salim et al. 2017), increasing this factor reduces electricity consumption.

### Estimate results by productive sectors

Table 6 shows the estimates for the agricultural sector of Eq. (5). The obtained results show that the cube production coefficient is not significant, so a new re-estimation has been performed omitting this variable (model 1). Likewise, the HDD coefficient is not significant. Therefore, a new estimate has been performed omitting its squared value (model 2).

**Table 6:** Estimate results for the agriculture sector

Variable	Coefficient	Model 1	Model 2
*Y*	*β*_1_	0.987***	1.008***
		(0.060)	(0.080)
*Y*^2^	*β*_2_	0.776***	0.780***
		(0.106)	(0.117)
*Y*^3^	*β*_3_		
*K*	*β*_4_	0.920***	0.933***
		(0.065)	(0.067)
*H*	*β*_5_	− 0.083***	− 0.139***
		(0.027)	(0.030)
*HDD*_18_	*β*_6_	0.073	− 0.036***
		(0.052)	(0.012)
*HDD*_18_^2^	*β*_7_	0.015**	
		(0.006)	
*CDD*_22_	*β*_8_	− 0.039**	− 0.040**
		(0.015)	(0.020)
*CDD*_22_^2^	*β*_9_	0.002	0.005*
		(0.002)	(0.002)

Standard error in parenthesis. *** denotes significance at the 1% level, ** at the 5% level, and * at the 10% level

Models 1 and 2 results show again that the relationship between production and electricity consumption is not linear. In these estimates, coefficients related to the production and its squared value are positive and significant. Therefore, a progressively increasing relationship is observed. Regarding the temperature variables, temperature increase above 22 °C tends to reduce electricity consumption. Nevertheless, as it continues to increase, this effect tends to disappear. On the other hand, temperature decreases below 18 °C tend to decrease electricity consumption (as shown in model 2 results). Nevertheless, there is a non-significant effect observed in model 1. Finally, it is worth noting that the results related to the productive factor are quite similar to those observed for total productive sectors. A positive and negative result of physical capital and human capital is observed respectively.

The results obtained for this sector should be interpreted with caution, since some other factors may influence the sector’s production, while other climatic conditions, such as rainfall or droughts, may have an effect on energy consumption, mainly due to their influence on irrigation.

Table 7 shows the estimates of Eq. (5) for the construction sector. The results obtained show that the squared and cubed production coefficients are not significant. Likewise, the squared variable coefficient is not significant when the cube variable is omitted in a new estimate. Therefore, it is not possible to affirm that there is a non-linear relationship between production and electricity consumption in the construction sector. Thus, a new estimation has been performed omitting these variables. These new estimate results are shown in Table 7, in the column named model 1. Additionally, since the temperature variables’ coefficients are not significant, the previous model has been re-estimated omitting the respective squared. These results are shown in model 2 of Table 7. It should be noted that the variables’ elimination does not significantly change the estimated value for the rest of the variables. Likewise, if the temperature variables are excluded from the model, no relevant changes are observed in the rest of the estimated coefficients.

**Table 7:** Estimate results for the construction sector

Variable	Coefficient	Model 1	Model 2
*Y*	*β*_1_	0.870**	0.878***
		(0.266)	(0.223)
*Y*^2^	*β*_2_		
*Y*^3^	*β*_3_		
*K*	*β*_4_	1.064***	0.884***
		(0.156)	(0.125)
*H*	*β*_5_	− 0.151**	− 0.227***
		(0.070)	(0.065)
*HDD*_18_	*β*_6_	− 0.006	0.030
		(0.090)	(0.040)
*HDD*_18_^2^	*β*_7_	− 0.007	
		(0.012)	
*CDD*_22_	*β*_8_	− 0.006	0.001
		(0.020)	(0.009)
*CDD*_22_^2^	*β*_9_	0.001	
		(0.003)	

Standard error in parenthesis. *** denotes significance at the 1% level, ** at the 5% level, and * at the 10% level

Models 1 and 2 show a positive linear relationship between the variables. As the production increases in the construction sector, the electricity consumption also does. Additionally, results show, as in previous estimates, that a greater physical capital use contributes positively to electricity consumption, while human capital reduces it. Finally, with respect to temperatures, it should be noted that none of the estimates reflects a significant effect of these variables.

Table 8 shows the estimate results for the industrial sector. Once again, the estimation of Eq. (5) shows non-significant values for the cube production coefficient. Therefore, this variable has been eliminated and the equation re-estimated. The results are presented in the third column of Table 8 (model 1). In addition, as the temperature variables’ coefficients are not significant, the squared HDD and CDD variables have been omitted in a new estimate. The new results are shown in the last column (model 2) of Table 8. The results do not significantly change when the aforementioned variables are eliminated.

**Table 8:** Estimate results for the industrial sector

Variable	Coefficient	Model 1	Model 2
*Y*	*β*_1_	1.080***	0.919***
		(0.168)	(0.149)
*Y* ^2^	*β*_2_	− 0.795**	− 0.769**
		(0.395)	(0.316)
*Y *^3^	*β*_3_		
*K*	*β*_4_	0.422**	0.355**
		(0.157)	(0.104)
*H*	*β*_5_	0.021	0.015
		(0.045)	(0.040)
*HDD*_18_	*β*_6_	0.062	0.021*
		(0.045)	(0.012)
*HDD*_18_^2^	*β*_7_	0.004	
		(0.005)	
*CDD*_22_	*β*_8_	0.004	0.001
		(0.010)	(0.009)
*CDD*_22_^2^	*β*_9_	0.001	
		(0.001)	

Standard error in parenthesis. *** denotes significance at the 1% level, ** at the 5% level, and * at the 10% level

Models 1 and 2 show a U-shaped inverted relationship between production and electricity consumption. Therefore, evidence favorable to the energy-EKC hypothesis for the industrial sector is found. In this sense, it should be noted that the industrial sector has had, at a European level, very significant energy intensity improvements, with many of its subsectors having achieved greater energy efficiency improvements (Schulze et al. 2016). Related to temperature variables, the results indicate that there is no clear temperature effect on electricity consumption in this sector. There is only some evidence in favor of the theory that a drop in temperatures below 18 °C (increase HDD) can lead to an increase in electricity consumption. The lack of sensitivity of the sector to variations in temperature may be associated with the fact that this sector is very heterogeneous, with very specific production processes. Finally, once again, a significant positive effect of physical capital on electricity consumption is observed. Meanwhile, human capital does not have a significant effect on it.

Table 9 shows the estimate results for the public administration sector. As shown in the third column (model 1), the result of Eq. (5) estimate shows a non-significant coefficient of the cubed production variable, with the rest of the estimated coefficients being significant. Omitting that variable from the equation, the re-estimated coefficients maintain quite the same values and remain significant (model 2).

**Table 9:** Estimate results for the public administration sector

Variable	Coefficient	Model 1	Model 2	Model 3
*Y*	*β*_1_	1.252***	1.169***	0.921***
		(0.360)	(0.342)	(0.206)
*Y* ^2^	*β*_2_	5.042**	4.698**	3.845**
		(2.341)	(2.305)	(1.582)
*Y *^3^	*β*_3_	− 0.192		
		(2.273)		
*K*	*β*_4_	1.129***	1.124***	0.920***
		(0.111)	(0.118)	(0.161)
*H*	*β*_5_	0.168***	0.203***	0.136***
		(0.027)	(0.036)	(0.027)
*HDD*_18_	*β*_6_	− 0.184***	− 0.118***	0.002
		(0.031)	(0.041)	(0.005)
*HDD*_18_^2^	*β*_7_	− 0.025***	− 0.017***	
		(0.004)	0.005	
*CDD*_22_	*β*_8_	0.036***	0.055***	0.055***
		(0.010)	(0.012)	(0.010)
*CDD*_22_^2^	*β*_9_	0.018***	0.019***	
		(0.00)	(0.002)	

Standard error in parenthesis. *** denotes significance at the 1% level, ** at the 5% level, and * at the 10% level

Both models’ results show a clearly progressively increasing effect of electricity consumption with respect to production, so that the energy-EKC hypothesis cannot be verified. There is also a positive effect of the increase in temperatures above 22 °C (CDD increase) on electricity consumption. This effect is also progressively increasing. With regard to the increase of temperatures below 18 °C (HDD increase), a negative effect is observed once again. As before, Eq. (5) is re-estimated to test the model without considering the squared temperature coefficients. As in Table 5, the coefficient related to the CDD is still positive and significant, but in this time, the coefficient related to the HDD variable becomes positive but not significant. Therefore, it may be explained by a fuel substitution effect, as lower temperatures are registered in the continental climate zone, where buildings mostly do not use electricity for heating.

The results also show that both physical and human capital cause electricity consumption increase. Nevertheless, the growth experienced by the increase in physical capital is much greater than that caused by human capital. The human capital coefficient’s positive sign may suggest that the activities carried out in the sector by more qualified personnel require greater use of information and communication technology (ICT) devices and services. In this regard, it should be noted that, according to Van Heddeghem et al. (2014), worldwide ICT electricity consumption growth is higher than worldwide electricity consumption growth.

Finally, Table 10 shows the results of the estimation of Eq. (5) for the service sector. As this estimate does not show significant values for the squared and cubic production variable (model 1), this equation has been re-estimated without considering them. The new results are shown in the fourth column (model 2). Additionally, in the last column, the values of re-estimating the above equation omitting the CDD squared variable are shown (model 3). The results are quite similar to those obtained previously.

**Table 10:** Estimate results for the service sector

Variable	Coefficient	Model 1	Model 2	Model 3
*Y*	*β*_1_	1.547***	1.766***	1.754***
		(0.127)	(0.086)	(0.086)
*Y*^2^	*β*_2_	0.342		
		(0.337)		
*Y*^3^	*β*_3_	2.924		
		(1.823)		
*K*	*β*_4_	0.016	0.181**	0.179**
		(0.076)	(0.090)	(0.090)
*H*	*β*_5_	− 0.092***	− 0.062**	− 0.063**
		(0.028)	(0.027)	(0.027)
*HDD*_18_	*β*_6_	− 0.354***	− 0.367***	− 0.361***
		(0.021)	(0.020)	(0.020)
*HDD*_18_^2^	*β*_7_	− 0.046***	− 0.046***	− 0.045***
		(0.002)	(0.002)	(0.002)
*CDD*_22_	*β*_8_	0.020***	0.016***	0.021***
		(0.005)	(0.006)	(0.002)
*CDD*_22_^2^	*β*_9_	0.001	− 0.000	
		(0.000)	(0.000)	

Standard error in parenthesis *** denotes significance at the 1% level, ** at the 5% level, and * at the 10% level

These results indicate that there is a positive linear relationship between electricity consumption and production. It is worth noting that the coefficient linked to the production is higher than those obtained for the other sectors, showing that this sector is a high electricity consumer. Therefore, the energy measures in this sector are relevant to control for energy efficiency. Likewise, it is observed, as in the previous studies, that there is a positive effect of temperatures above 22 °C, and a negative effect of temperatures below 18 °C, on electricity consumption. Finally, a positive effect of physical capital and a negative effect of human capital are observed once more.

## Discussion

Some considerations can be made related to the obtained results of previous estimates. Firstly, it should be noted that there are important sectoral differences in the production effect on electricity consumption. Thus, while there is a linear relationship between both variables in the construction and service sectors, and a progressively increasing relationship in the agricultural and public administration ones, an inverted U-shaped relationship, consistent with the energy-EKC hypothesis, is observed in the industrial sector. In relation to these results, two aspects should be highlighted. On the one hand, these results are in line with previous studies’ results (Costantini and Martini 2010; Zhang and Xu 2012), which emphasize the need to carry out sectoral studies when analyzing the effect of income or production on energy consumption. In this sense, these differences suggest the need to carry out different energy policies according to the economic sector. On the other hand, the results show that the only sector that presents an inverted U-shape is the industrial one; this sector, according to Schulze et al. (2016), is the one that has intensively promoted energy efficiency improvement in recent years. Thereby, it may be convenient to deepen these measures in other sectors, especially in those that show a progressively growing relationship and in the service sector, where there is a linear relationship, but its elasticity is very high.

Secondly, the results show a positive and growing effect of temperatures above 22 °C on electricity consumption, in the total economy and in the sectors belonging to the tertiary one. In this sense, global warming, and the Spanish economy tertiarization trend can boost greater electricity consumption in the country. In this sense, as stated in Li et al. (2018), the hotter summer would impact larger in the electricity increase consumption than the colder winter. The negative effect of temperatures below 18 °C may also suggest that the decrease of heating degree days (HDD decrease) may have negative effects on electricity consumption, which could be caused by fuel and gas changes to electricity. In this sense, Li et al. (2012) stated that increasing temperatures could have important implications for the electricity consumption, as heating is provided largely by oil and gas boiler plants whereas cooling mainly relies on electricity. It is also worth noting that Spain has 3 main different climatic zones, where the heating systems may present significant differences related to the use of electricity for heat.

Thirdly, it is also important to note the significant differences in the effect of temperature on electricity consumption by the economic sector. Thus, while the construction and industrial sectors do not seem to be particularly sensitive to temperatures, the service and agriculture sectors are significantly affected by them. These results are in line with those obtained by Fan et al. (2015). In their study, they show that the most sensitive sector to temperatures is the service one, which has a similar behavior to the residential sector. Therefore, it can be considered that the main effect of temperatures on the service sector is related to the need to keep buildings adequately acclimatized. In this sense, the positive effect of high temperatures is related to the penetration of air conditioning in the hottest areas (Silva et al. 2020). The low effect of temperatures in the secondary sector, construction, and industrial, is in line with the results obtained by Moral-Carcedo and Pérez-García (2015), which show the absence of a significant effect of temperatures in this sector, except for the manufacturing of food products sector. The authors justify its temperature sensitivity to the refrigeration use.

Fourthly, the way in which production is carried out also seems to affect electricity consumption. On the one hand, it is observed that the production capitalization tends to generate greater consumption of electricity. On the other hand, the use of more human capital tends to decrease (with the exception of the public administration sector) electricity consumption. These results are in line with previous studies, such as Fang and Chen (2017), Salim et al. (2017), and Chen and Fang (2018). Results may also suggest the convenience of conducting studies distinguishing between types of capital, especially among those associated with new ICT.

Finally, it should be noted that the results obtained show that economic growth, rising temperatures, and capitalization have a positive effect on electricity consumption. Thus, in the future, it is foreseeable that there will be a growth in the demand for electricity in Spain. In this case, it may be appropriate for the grid to be expanded and adapted to these new needs. Likewise, in a context of rising natural gas prices, it may be advisable to promote the use of alternative energies to meet the growth in electricity demand. Along these lines, it may be appropriate to adapt the grid to support the installation of new renewable energy facilities, but the need to resort to alternative sources, such as nuclear energy, may also be considered.

## Conclusion and policy implications

The energy consumption analysis has been established at the center of the political and economic debate on climate change and global warming in recent years. Of all the energy sectors, the electricity sector has special relevance. Therefore, the study of the electricity consumption behavior is necessary to promote adequate policies to achieve the Paris targets.

This paper analyzes the effect of certain factors on the electricity consumption in Spain. This paper estimates, by using panel data, an electricity consumption function that depends on GVA, production factors such as capitalization, and the use of human capital and temperatures. The function is estimated for total productive electricity consumption and for the agricultural, construction, industrial, service, and public administration sectors. The data panel refers to the 17 Autonomous Communities of Spain for the 2000–2013 period.

The results show that there are important sectoral differences in the effect that GVA has on electricity consumption. A linear relationship is observed in the construction and service sectors, a progressively increasing relationship in the agricultural and public administration sectors, and an inverted U-shaped relationship in the industrial one. For a total productive economy, the relationship is N-shaped.

The results also indicate a positive and increasing effect of temperatures above 22 °C (CDD) on electricity consumption in the total economy and in the tertiary sector. On the contrary, results indicate a negative effect of temperatures below 18 °C (HDD) in all sectors, which could be motivated by fuel changes and/or significant differences in the heating systems by climatic zones. These results may indicate that global warming may induce an electricity demand growth in Spain, especially related to the cooling needs.

In addition, the results show that the productive model affects electricity consumption. Capitalization has a positive effect on electricity consumption in all sectors, while human capital has negative effects, except for the public administration sector.

Finally, it should be considered that, given that economic growth, global warming, and capitalization tend to generate greater electricity consumption, it may be appropriate to carry out policies that mitigate this consumption growth. Among these policies, it may be appropriate to promote energy efficiency policies (especially in non-industrial sectors) and human capital investments. These policies should be designed according to the economic sector. In addition, since it is foreseeable that the demand for electricity will grow in Spain, for the reasons mentioned above, it is advisable that the country prepares itself to meet this growth in demand. To this end, it is considered appropriate to extend the electricity grid. Likewise, the grid must be adapted to be able to support the installation of new renewable energy plants. Finally, it is necessary to rethink the use of alternative energy sources for electricity generation, such as nuclear energy.

## Data Availability

The datasets used and/or analyzed during the current study are available from the corresponding author on reasonable request.
